# The SIAMESE family of cell-cycle inhibitors in the response of plants to environmental stresses

**DOI:** 10.3389/fpls.2024.1362460

**Published:** 2024-02-16

**Authors:** Jeanne Braat, Michel Havaux

**Affiliations:** Aix Marseille University, CEA, CNRS UMR7265, Bioscience and Biotechnology Institute of Aix Marseille, Saint-Paul-lez-Durance, France

**Keywords:** cell cycle, environmental stress, SIAMESE (SIM), SIAMESE-RELATED (SMR), apocarotenoids, plant stress tolerance

## Abstract

Environmental abiotic constraints are known to reduce plant growth. This effect is largely due to the inhibition of cell division in the leaf and root meristems caused by perturbations of the cell cycle machinery. Progression of the cell cycle is regulated by CDK kinases whose phosphorylation activities are dependent on cyclin proteins. Recent results have emphasized the role of inhibitors of the cyclin-CDK complexes in the impairment of the cell cycle and the resulting growth inhibition under environmental constraints. Those cyclin-CDK inhibitors (CKIs) include the KRP and SIAMESE families of proteins. This review presents the current knowledge on how CKIs respond to environmental changes and on the role played by one subclass of CKIs, the SIAMESE RELATED proteins (SMRs), in the tolerance of plants to abiotic stresses. The SMRs could play a central role in adjusting the balance between growth and stress defenses in plants exposed to environmental stresses.

## 1 Introduction

When exposed to environmental stress, plants reduce their vegetative growth, hence conserving and redistributing resources and increasing their chance of survival (Mareri et al., 2022). Plant organ growth relies on two main phenomena: cell proliferation and cell expansion (Powell and Lenhard, 2012). The coordination of these two processes during leaf and root growth ultimately determines leaf/root size and shape. In specialized zones of leaf and root tissues called meristems, growth is driven exclusively by cell proliferation. When cells leave the meristematic zone, they begin to exit the cell cycle and undergo differentiation that is accompanied by increases in cell size. Biotic and abiotic stress stimuli can negatively affect both cell division and cell expansion (Alves and Setter, 2004; Qi and Zhang, 2020; Shimotohno et al., 2021). In the meristems, stress-induced inhibition of cell proliferation occurs through perturbations of the cell cycle machinery (Shimotohno et al., 2021).

The regulation of cell cycle transitions in plants is similar to that of animals (Dewitt and Murray, 2003; Inzé and De Veylder, 2006). Transitions between stages of the cell cycle are controlled by a class of Ser/Thr kinases known as cyclin-dependent kinases (CDKs). As indicated by their name, the kinase activity of CDKs depends on their interaction with regulatory cyclin (CYC) proteins. Cell cycle progression is regulated by periodic expression of CYCs and their ubiquitin-mediated proteolysis and by phosphorylation of a variety of targets by CYC/CDK complexes. However, cell cycle progression is also regulated by a panoply of inhibitors (CKIs) of CYC/CDK constituted by the KIP-related proteins (KRPs) and the SIAMESE family (SIM and SIAMESE RELATED SMRs) (Kumar and Larkin, 2017). It is generally believed that stress-induced cell cycle inhibition is principally mediated by transcriptional upregulation of those CKIs, leading to cell proliferation arrest and plant growth inhibition. Here, we review this scenario, with a particular emphasis on the involvement of SMRs in the response of plants to abiotic stresses and in the modulation of their stress tolerance.

## 2 The cell cycle

To facilitate understanding of the link between CKIs and the responses of plants to environmental changes, this review will first present some general aspects of the cell cycle and its regulation. More detailed descriptions of the plant cell cycle can be found in several previous reviews (*e.g*. Mironov et al., 1999; Dewitt and Murray, 2003; Inzé and De Veylder, 2006; Lang and Schnittger, 2020; Sablowski and Gutierrez, 2022). The cell cycle in eukaryotes generally follows the same fundamental pattern across all species: It is divided into four main phases, including the synthesis (S) and mitosis (M) phases, which lead to the duplication of the genome and its distribution into daughter cells, respectively. S and M are separated by the pre-replicative Gap phase 1 (G1) and the post-replicative Gap phase 2 (G2). During G1, the actively growing cell synthesizes proteins and organelles necessary for cell division. Then the cell reaches a checkpoint where it assesses environmental conditions, cell size, and DNA integrity before entering the S phase. Once the DNA is replicated, the cell enters the G2 phase, during which cell growth continues, and proteins are synthesized in preparation for mitosis. Eventually, the mother cell completes its cell cycle by entering the mitotic phase, resulting in two daughter cells that can differentiate, enter senescence or undergo further division. Plant cells are embedded in a pecto-cellulosic wall and hence the cytoplasmic division (cytokinesis) cannot occur through constriction, as in the case of animal cells. Plant cytokinesis is directed by an organelle called the phragmoplast, an array of microtubules partially embedded in a protein matrix along which Golgi vesicles carry the raw materials to synthesize a new cell plate (Smith, 2002; Jürgens, 2005). The cell plate is a cellulose-rich structure that forms at the center of the cell and is the precursor to the definitive cell wall of the two daughter cells.

Coordination between cell division and growth is required for proper development of multicellular organisms. In response to this need, complex systems of cell cycle regulation have evolved in plants and animals. The proper progression of the cell cycle is tightly controlled by a family of regulatory Ser/Thr kinases known as ‘cyclin-dependent kinases’ (CDK) (Mironov et al., 1999; Joubès et al., 2000). As kinases, the role of CDK is to phosphorylate targeted proteins specific to different phases of the cell cycle by activating or deactivating them, and thereby causing the unidirectional progression of the cycle. Plant CDKs have been classified into eight different classes: CDKA–CDKG and the CDK-like kinases (CKL) (Joubès et al., 2000; Vandepoele et al., 2002). Among them, CDKBs are specific to plants and, along with CDKAs, they have a primary function in the cell cycle control (Inzé and De Veylder, 2006). CDKA is considered as a functional ortholog to yeast CDC2/CDC28 and mammalian CDK1, playing a crucial role in facilitating transitions from G1 to S and G2 to M phases in the cell cycle. On the other hand, the two types of CDKB, CDKB1 and CDKB2, are exclusively expressed during the late S/M and G2/M phases, respectively (Magyar et al., 1997; Umeda et al., 1999).

Changes in structural conformation are necessary for the activation of CDKs. CDKs are consistently expressed throughout the cycle, and as their name suggests, the oscillations in their activities depend on corresponding oscillations in the levels of protein subunits known as cyclins. Cyclins are divided in two groups: transcriptional cyclins and cell cycle cyclins also known as canonical cyclins (Quandt et al., 2020). In plants, this last group is composed of A-, B- and D-type cyclins that have been well characterized (De Veylder et al., 2011; Van Leene et al., 2011). Cyclins A and B have been described as mitotic cyclins, preparing and regulating entry into mitosis. Cyclins D, on the other hand, prepare and control entry into the S phase. In Arabidopsis, there are at least 31 of these cyclins (10 cyclins A, 11 cyclins B, 10 cyclins D) (Vandepoele et al., 2002). However, other cyclins have been identified in Arabidopsis (C, H, L, P, T, U-type and SOLO DANCERS cyclins) some of which like CYCH;1 have functions in core cell cycle machinery (Wang et al., 2004; Van Leene et al., 2011).

By binding to CDKs, cyclins enable the displacement of the T-loop blocking the active site of the kinase (Gould et al., 1991; Russo et al., 1996). Throughout the cell cycle, the abundance of different cyclins is oscillating leading to the formation of a series of cyclin-CDK complexes (Morgan, 2007). These proteins are regulated not only at the transcriptional level but also through rapid and specific proteolysis by the ubiquitin 26S proteasome-dependent pathway. The full activation of CDK not only requires cyclin binding but also CDK phosphorylation mediated by cyclin-activating kinases (CAK), with four such kinases in Arabidopsis (CDKD;1-3, CDKF;1) (Umeda et al., 2005). CAK phosphorylates the complex and alters its conformation, triggering an affinity for its substrates.

The cell cycle is also regulated by CAK antagonistic proteins known as CDK inhibitors (CKI) (Kumar and Larkin, 2017). CKIs inactivate cyclin-CDK complexes either by binding to the CDK subunit and thereby modifying its conformation, or upstream of complex formation, by binding directly to the cyclin binding site (Sherr and Roberts, 1999). There are two CKI families in plants: the KIP-related proteins (KRPs) and the SIAMESE family (SIM and SIAMESE RELATED SMRs). Under certain conditions, CKIs can trigger a modified cell cycle process called endoreplication or endoreduplication. Endoreplication is a variation of the eukaryotic cell cycle which commonly appears in plants, in which the stages of mitosis and cytokinesis are bypassed in favor of repeated DNA replication, resulting in a polyploid cell (De Veylder et al., 2011). Endoreplication occurs at multiple instances during plant development and is often associated with differentiating cells that have already exited the mitotic cycle. This is why trichome cells in *Arabidopsis thaliana* are commonly used as a model to study endoreplication (Walker et al., 2000; Schnittger and Hülskamp, 2002). For example, endoreplication occurs during the formation of endosperm in wheat grains or during the formation of the pericarp in tomato fruits (Sabelli and Larkins, 2009; Chevalier et al., 2011).

Endoreplication in plants occurs also in response to biotic and abiotic stresses. This process is seen as a specific pathway of controlling gene expression: increasing the number of copies of genes allow the plants to increase the copies of genes necessary for passive and active defense against biotic and abiotic stresses. Thus endoreplication can help plants to survive in a changing and often very unfavorable environment (Kołodziejczyk et al., 2023).

Although the molecular mechanisms initiating this process are increasingly understood especially due to the use of the model organism *Arabidopsis thaliana* (De Veylder et al., 2002; Cook et al., 2013), the physiological roles of endoreplication remain to be clarified: often, endoreplication is positively associated with cell growth and differentiation (Melaragno et al., 1993; Roeder et al., 2010; Massonnet et al., 2011). In other cases, the relationship between high polyploidy and increased cell growth is not confirmed (Cookson and Granier, 2006; De Veylder et al., 2011; Tsukaya, 2019).

## 3 SMRs and other inhibitors of the cell cycle

KRP and SMR are low-molecular-weight proteins with a size ranging from 8 to 19 kDa for SIM/SMR proteins and from 19 to 32 kDa for KRP proteins. The KRP gene family was initially discovered based on sequence similarities with mammal KIP genes, and the SMR gene family was identified based on the mutant phenotype of the *SIM* gene, which results in trichomes that divide instead of endoreplicating (Walker et al., 2000).

**Figure 1:**
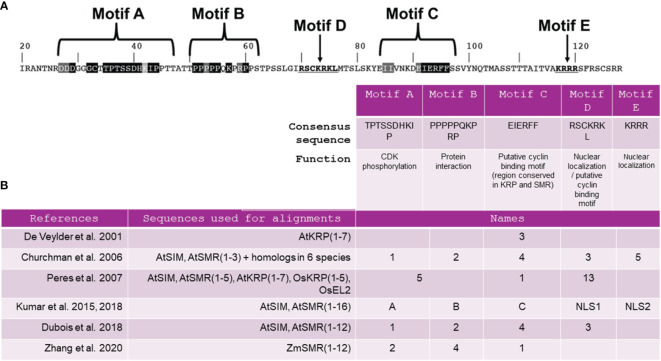
Presentation of the five major motifs previously identified in SIM/SMR sequences. **(A)** SIM amino acid sequence, starting at residue 20, indicating the location of the motifs and their consensus sequences adapted from Kumar et al. (2018). **(B)** Nomenclature assigned to these motifs according to findings from 6 comprehensive functional analyses of SIM/SMR sequences. At, *Arabidopsis thaliana;* Os*, Oryza sativa;* Zm*, Zea mays.*.

Primary sequence alignments of SMR and KRP proteins among several species have allowed the identification of a unique conserved domain thought to be a cyclin-binding motif named motif C (Churchman et al., 2006; Peres et al., 2007; Kumar et al., 2015). The consensus sequence for motif C is EIERFF (**Figure 1B**). Motif C is described as a domain of the rice SMR EL2 protein that interacts with D-cyclin (Peres et al., 2007) and is also a domain of the KRP1 protein that interacts with CYCD3;1 (Wang et al., 1998; Churchman et al., 2006). However, recent findings suggest that motif C may not be universally critical for every CKI interaction. In addition to motif C, SIM and SMR share two other conserved domains, commonly referred as motifs A and B (**Figure 1A**). Kumar et al. (2018) have demonstrated that mutations within SIM motif A disrupted CDKA;1 binding, while mutations in motifs B and C have minimal impact on the ability of SIM to bind CDKA;1. Furthermore, a recent study has indicated that the same region within motif A can also function as a CYCA2:3 binding motif, challenging the notion that motif C is indispensable for binding to this cyclin (Wang et al., 2020). This suggests that SMRs establish interactions with cyclins not only through motif C but also through motif A, and the relative importance of these motifs may vary among different cyclin/SMR complexes.

Essential motifs other than A, B and C play a critical role in the comprehensive functionality of SIM/SMR. Initially designated as a cyclin-binding motif (Churchman et al., 2006), motif D in **Figure 1** was later found to have no impact on SIM function when mutated within the SIM protein (Kumar et al., 2015). Meanwhile, motifs 3 and 5, previously identified by Churchman et al. (2006), have been redefined as NUCLEAR LOCALIZATION SIGNAL 1 (NLS1) and NUCLEAR LOCALIZATION SIGNAL 2 (NLS2) (Kumar et al., 2018) (**Figure 1B**). A construct lacking motif C, NLS1 and NLS2 was unable to complement the *sim* mutant, suggesting that at least NLS1 or NLS2 is required for the biological function and complete nuclear localization of SIM. A study by Dubois et al. (2018) emphasized the significance of the C-terminal segment of the SMR1 protein, containing motif C, NLS1 and NLS2, in the nuclear localization of SMR1. However, uncertainties persist regarding these motifs. Specifically, it appears that the NLSs in SIM may serve more specific functions than targeting the protein to the nucleus (Kumar et al., 2018). Furthermore, the authors suggest that additional C-terminal sequences within the protein might also play a role in localizing SIM to the nucleus or in modifying protein stability.

SIM/SMR are found in a wide range of land plant lineages, including bryophytes, lycophytes, dicots and monocots (Kumar et al., 2015). Thus, orthologs of the Arabidopsis At-SIM in the monocotyledonous plant *Oryza sativa* have been identified under the names EL2 and EL2-like (Peres et al., 2007). SIM/SMR homologs are also known in *Solanum lycopersicum* (Sl-SMR1, Sl-SMR2, Sl-SIP4), *Solanum tuberosum* (St-SMR1), *Populus tremula* (Pt-SMR1), and *Glycine max* (Gm-SMR1) (Churchman et al., 2006). Orthologs of KRP are found at least in 40 plant species, including monocots (*Oryza sativa*, OS-KRP1 to 6) and dicots (*Solanum lycopersicum*, Sl-KRP1 and 2, *Populus tremula*, Pt-ICK1 to 6) and also in 2 gymnosperms. There are no such homologues for *KRP* and *SMR* genes in algae (*Chlamydomonas reinhardtii* and *Ostereococcus tauri).*

*Arabidopsis thaliana* has 21 CKIs comprising 7 members of the ICK/KRP family (KRP1 to 7) (Wang et al., 1997; De Veylder et al., 2001; Wang et al., 2006), and 17 members of the SIM/SMR family: SIAMESE (SIM, Walker et al., 2000), the SIAMESE RELATED (SMR) from 1 to 13 and the putative SMR14, SMR15 and SMR16 (Churchman et al., 2006; Van Leene et al., 2010; Kumar et al., 2015). While numerous studies address the structure, function, and regulation of KRP proteins in the cell cycle and plant development, the structural and functional characterization, as well as the regulation of SIM/SMR proteins, are still in their early stages of investigation.

Several interactomic approaches based on the techniques of yeast two-hybrid, Bimolecular Fluorescence Complementation (BiFC) and Tandem Affinity Purification (TAP) provide an overview and an exhaustive list of 416 interactions detected among key regulators of the cell cycle in Arabidopsis, including 31 involving KRP (Van Leene et al., 2011). The other interactomic study presents 119 interactions between KRP and key cell cycle regulators (Boruc et al., 2010). The KRP family, belonging to the core machinery of the cell cycle, exhibits diverse functions in regulating cell number and size and more broadly in organ and plant growth (Gonzalez et al., 2012). Notably, KRP5 has been shown to be a multifunctional protein bridging cell elongation and endoreplication (Jégu et al., 2013). KRP5 interacts with CYCD3;1 leading to inhibition of CYCD/CDKA complexes. KRP5 can also directly bind to chromatin and induce gene transcription involved in endoreplication, such as CDC20, encoding an activator of APC/C. KRP5 can also decondense heterochromatin (Jégu et al., 2013). Another study showed that KRP4 is critical for correcting cell size variability in the shoot stem cell niche by adjusting the G1 phase duration before DNA synthesis in the S phase (D’Ario et al., 2021).

Regulation of CKIs is believed to primarily take place post-transcriptionally, with secondary regulatory mechanisms operating at the transcriptional level (Liu et al., 2008; Ren et al., 2008; Jun et al., 2013; Kumar and Larkin, 2017; Guo et al., 2023). It is probable that SMRs undergo post-transcriptional activation following the initiation of the S phase. Thus, these genes mostly interact with G2/M-phase cyclins and CDK in Arabidopsis (Walker et al., 2000; Churchman et al., 2006), suppressing the mitotic step in favor of endoreplication. Similarly, the rice SMR protein, EL2, also interacts with cyclins D and CDKA;1 (Peres et al., 2007).

**Figure 2:**
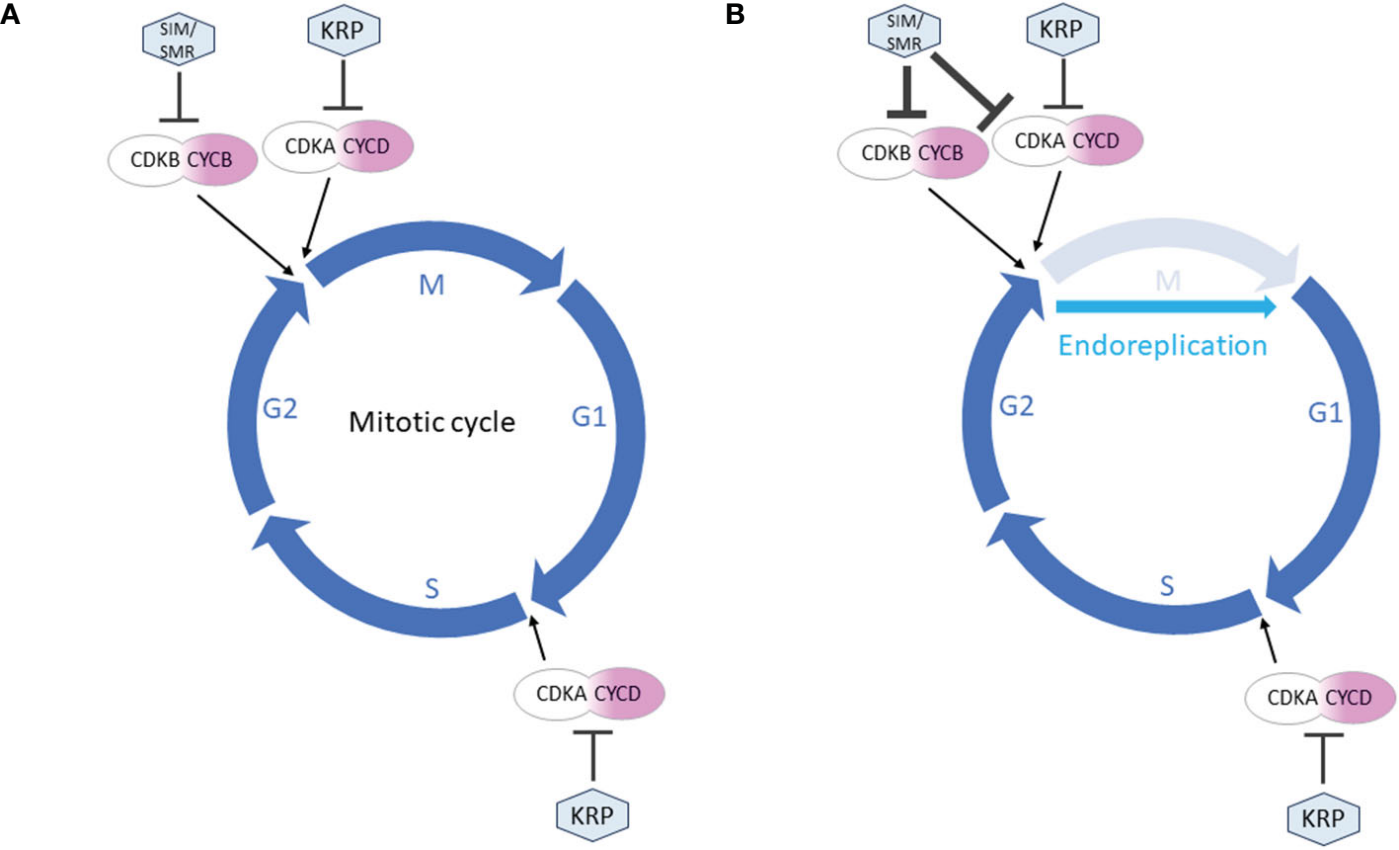
Simplified roles of SMR and KRP in the cell cycle regulation. The mitotic cell cycle proceeds through 4 phases: M (mitosis), G1 (gap 1), S (synthesis) and G2 (gap 2). **(A)** During mitotic cycle, KRPs primarily inhibit CDKA;1, the main G1/S CDK. Thus KRP can block entry into both S and M phase. SMRs are inactive throughout G1 and G1/S transition allowing S phase to proceed. They can block entry into M-phase only. **(B)** During endoreplication, the cell cycle skips the M phase. KRP activity continues unchanged in establishing G1 checkpoint. In G2 phase, the increased expression of SIM/SMR strongly inhibits M phase, resulting in a switch from mitotic to endoreplication cycles.

While both SMR and KRP function as inhibitors of CDK and cell division, they fulfill distinct roles in the cell cycle. It is generally believed that KRPs and SMRs contribute to the establishment of cell cycle checkpoints in G1 and G2, respectively (**Figure 2**). More detailed descriptions of the differences between SMR and KRP roles in cell cycle can be found in Kumar and Larkin (2017). KRPs are primarily inhibitors of the CDKA;1 activity, the main Arabidopsis G1/S CDK.

Modification of CKI levels strongly perturbs the plant phenotype: mild overexpression of *KRP1* and *KRP2* in mitotically active cells induces the onset of endoreplication resulting in a slight reduction in leaf size. Conversely, high expression levels of *KRP1* and *KRP2* in transgenic plants hinder both DNA replication and mitosis resulting in small serrated leaves and, in some cases, cell death (Wang et al., 2000; De Veylder et al., 2001; Schnittger et al., 2003; Verkest et al., 2005). In contrast, strong overexpression of SIM/SMR has never been observed to inhibit DNA replication or cause cell death (Churchman et al., 2006; Kumar et al., 2015). High *SMR4* or *SMR5* overexpression also leads to serrated leaves and in some cases in leaf biomass reduction (Yi et al., 2014) but in other cases not (Braat et al., 2023).

Regarding the mutants, single loss-of-function SMR or KRP mutants often lead to no phenotype which is commonly attributed to the redundant functionality of SMR and KRP or a potential compensatory mechanism between the gene family members (Cheng et al., 2013; Sizani et al., 2019; Nomoto et al., 2022). However, multiple loss-of-function CKI mutants have various effects on plant growth. *sim/smr1/smr2* and *krp4/6/7* triple mutations lead to increased leaf area (Kumar et al., 2015; Sizani et al., 2019) whereas *smr1/smr2/smr13*, *smr1/smr2/smr9/smr13* and *smr5/smr7/smr4* did not exhibit apparent phenotypes regarding the overall plant growth (Hendrix et al., 2020; Yamada et al., 2022). The early-stage growth of *krp1/2/5/6/7* is accelerated when cultivated in Petri dishes, but this enhancement gradually diminishes when the plants are transferred in soil (Cheng et al., 2013). These findings suggest that the phenotype of KRP mutants is influenced by the growth conditions. Similar observations were noted in SMR-overexpressing lines, which displayed contrasting root length phenotypes depending on the growth conditions, whether *in vitro* or in soil (Braat et al., 2023).

## 4 CKIs and cell division under environmental stresses

**Figure 3:**
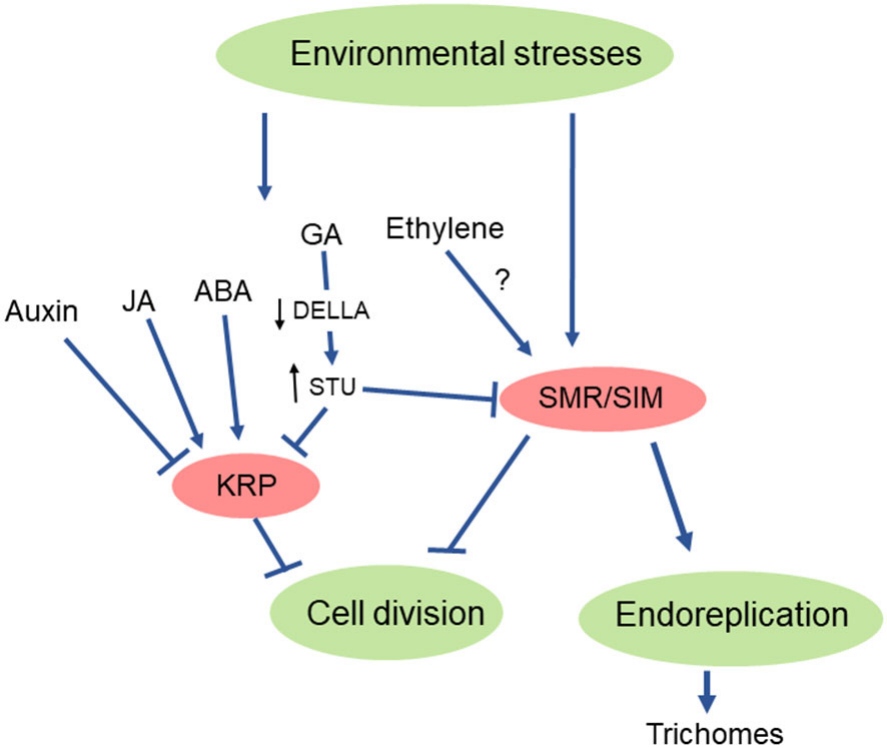
Scheme of the induction of CKIs by environmental stresses.

It is well known that adverse environments affect plant growth (Mareri et al., 2022). This may simply be because the environmental conditions are not optimal for growth. For instance, drought stress inhibits plant growth because water is needed for cell turgor that drives cell expansion (Ali et al., 2022). Cold stress reduces plant growth because metabolism is slowed down and enzyme activities are lower (Fürtauer et al., 2019). However, slower plant growth under stress is not only a passive consequence of the adverse environment. Plants also actively slow their growth by signaling mechanisms in response to stress (Shimotohno et al., 2021). As sessile organisms continuously exposed to changing environmental conditions, plants display extraordinary developmental plasticity to adjust their architecture to the environmental conditions. Proper organogenesis under changing environments is achieved by actively modulating the cell cycle machinery (Shimotohno et al., 2021). Multilevel regulation of CDK activities and their combinational capacities, as detailed above in chapters 2 and 3, appears to be particularly well designed for an efficient spatiotemporal control of the cell cycle in response to external signals. However, the knowledge on the participation and mode of action of CKIs in this process is still fragmented. The main observations on these aspects are reviewed below for the responses of plants to abiotic stresses and are summarized in **Figure 3**.

Phytohormones are central in the regulation of plant growth and development, and the concentration of phytohormones is affected by environmental stresses (Fahad et al., 2014; Waadt et al., 2022). However, information on the link between phytohormones and CKIs in the regulation of plant growth under environmental stresses is scarce. Within CKIs, the KRPs appear to be the most responsive to phytohormonal changes. Auxin, the cardinal phytohormone in plant growth regulation, has been shown to reduce the transcript levels of *KRP1* and *KRP2*, and this was associated with enhanced CDK activities (Himanen et al., 2002; Himanen et al., 2004; Sanz et al., 2011). On the opposite, *KRP1* and *KRP2* are highly expressed in the presence of an auxin transport inhibitor (Himanen et al., 2002; Himanen et al., 2004). *KRP2* overexpression decreases the number of lateral roots, as does auxin deficiency (Himanen et al., 2002; Himanen et al., 2004; Verkest et al., 2005). These observations indicate an inverse relationship between *KRP* expression and auxin concentration, consistently with the inhibitory effect of KRPs on plant growth.

Another class of phytohormones that regulates plant development and appears to interfere with CKIs are the gibberellins (GA). The Arabidopsis GA-deficient *ga1-3* mutant exhibits high expression of several genes encoding CKIs, and this effect is cancelled by GA (Achard et al., 2009). DELLA proteins constitute the link in the interaction between GA and CKIs. DELLA proteins are negative regulators of GA signaling (Achard et al., 2009), and GA promotes growth through cell expansion by stimulating destruction of DELLA proteins (Olszewski et al., 2002). DELLA enhances the expression of *KRP2* and *SIM* (Achard et al., 2009), and GA applications reduces their expression (Meguro and Sato, 2014). This suggests that GA signaling controls the cell cycle by suppressing KRP/SIM expression (Achard et al., 2009; Lee et al., 2012). STUNTED (STU) is an intermediate in this signaling process which conveys the signal through DELLA (Lee et al., 2012). Degradation of the DELLA repressor RGA by GA upregulates *STU* which in turn decreases the expression of genes coding for CKIs. The GA effects on growth are complex since they are also modulated by jasmonic acid. The latter hormone interferes with GA signaling by inducing DELLA proteins and delaying GA-mediated DELLA protein degradation, resulting in the induction of CKIs and causing cell cycle retardation (Cheng et al., 2011). On the other hand, the negative regulatory phytohormone ABA has also been reported to induce KRP expression in parallel with shoot growth inhibition in rice (Meguro and Sato, 2014). In Arabidopsis too, abscisic acid (ABA) triggers the expression of a gene (*ICK1*) of the *ICK/KRP* family (Wang et al., 1998). In contrast, ABA does not transcriptionally induce *SMR1*, although it appears to stimulate the turnover of the protein (Dubois et al., 2018). Based on the effects of the ethylene precursor on SMR1 levels, these authors suggested that ethylene could act upstream of *SMR1.* Accordingly, inhibition of cell proliferation in the Arabidopsis root meristem by ethylene has been shown in previous studies (Thomann et al., 2009; Street et al., 2015). Also, in young developing Arabidopsis leaves subjected to osmotic stress, ethylene negatively balances cell cycle progression via inhibition of CDK activity (Skirycz et al., 2011). Moreover, it should be noted that *SIM* and *SMR8* are targets of EIN3, a master regulator of the ethylene signaling pathway, in etiolated seedlings (Chang et al., 2013). The possible link between ethylene and SMR regulation would deserve to be further studied in the future.

While phytohormones modulate cell cycle inhibitors of the KRP family, SMR genes seem to be more directly responsive to the environmental conditions. For instance, stress-induced DNA damage is associated with the induction of *SMR* genes. DNA damage is sensed by two kinases, ATM and ATR (ATAXIA-TELANGIECTASIA-MUTATED and ATM and RAD3-RELATED), which phosphorylate and activate the transcription factor SOG1 (SUPPRESSOR OF GAMMA RESPONSE 1) (Yoshiyama et al., 2013; Hu et al., 2016). The latter regulator induces the expression of hundreds of genes involved in DNA repair and cell cycle arrest. In particular, SOG1 directly induces the genes for 3 CKIs: *KRP6*, *SMR5* and *SMR7* (Yi et al., 2014; Ogita et al., 2018). There are many reports describing transcriptional changes of *SIM/SMR* genes in response to environmental factors such as nitrate (Moreno et al., 2020), reactive oxygen species (Yi et al., 2014), drought (Dubois et al., 2018; Braat et al., 2023), DNA damage (Peres et al., 2007; Yi et al., 2014), cadmium pollution (Hendrix et al., 2018), low/high temperature (Peres et al., 2007; Zhang et al., 2020a) or salinity/osmotic stress (Peres et al., 2007; Zhang et al., 2020a). Interestingly, in those previous studies, *SMR5* was by far the most responsive *SMR* gene to the largest range of abiotic stresses (Peres et al., 2007; Dubois et al., 2018). Thus, SIM/SMR perceive environmental signals through transcriptional regulation, suppressing CDK activities and hence inhibiting cell division and promoting endoreplication.

**Figure 4:**
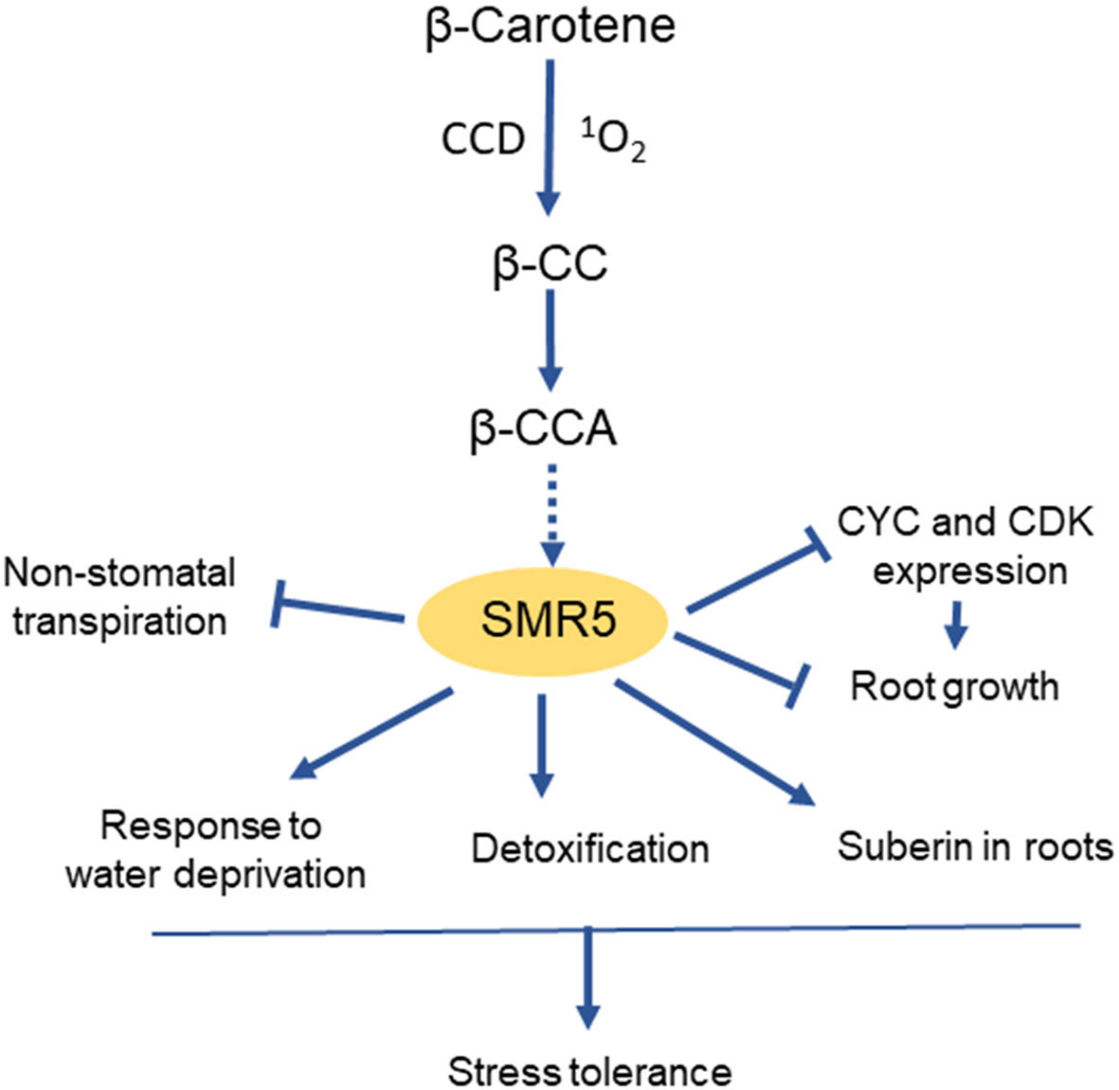
Induction of *SMR5* and its effects on plant growth and stress tolerance by the apocarotenoid β-CCA. CCD, carotenoid cleavage dioxygenase. β-CC, β-cyclocitral, precursor of β-CCA.

The exact mechanism by which *SMR* transcription is modulated by the abiotic stress conditions is still largely unknown. A recent work (Braat et al., 2023) has shown that *SMR5* is selectively induced by β-cyclocitric acid, an oxidation product of β-carotene that accumulates in plants exposed to climatic stresses such as drought or high light (D’Alessandro et al., 2019). Exposure of Arabidopsis seedlings to β-CCA concomitantly provoked a strong increase in *SMR5* expression and a down-regulation of many *CYC* and *CDK* genes (Braat et al., 2023). This compound was also found to inhibit root growth and to induce various genes of stress defense and cellular detoxification. Additionally, high expression levels of SMR5 were associated with the induction of several water-saving mechanisms such as suberin deposition in roots and decrease in non-stomatal transpiration (**Figure 4**). Thus, apocarotenoids could be molecular intermediates that link ROS production under environmental stresses and the adjustment of the balance between stress protection and growth through *SMR5* upregulation. The precursor of β-CCA, β-cyclocitral, is also a signaling apocarotenoid which also enhances drought tolerance (D’Alessandro et al., 2019). Similarly to β-CCA, β-cyclocitral acts independently of stomatal closure (Ramel et al., 2012) and inhibits root growth (Braat et al., 2023). In fact, β-cyclocitral is oxidized into β-CCA *in vivo*, and exogenous application of volatile β-cyclocitral on Arabidopsis seedlings resulted in a strong accumulation of β-CCA in leaves and roots (Braat et al., 2023). Although the effect of β-cyclocitral on SMR5 expression is not known, it is likely that the β-cyclocitral-induced phytoprotection against drought stress largely occurs through β-CCA. The receptor of the apocarotenoid molecule in this signaling pathway has not yet been identified and constitutes a major challenge for future research.

## 5 SMRs, new targets for improving plant tolerance to abiotic stresses?

SMR1 is induced by moderate water stress, possibly participating in the arrest of cell cycle progression and growth of the stressed plants (Dubois et al., 2018). Accordingly, overexpressing *SMR1* in Arabidopsis led to plant growth inhibition (Dubois et al., 2018). In fact, SMR1 is a short-lived protein, which is stabilized and accumulates under drought stress conditions. Interestingly, a recent study has revealed a specific role for SMR1 in the leaf epidermis (Dubois et al., 2023). SMR1 is required for differentiation of stomatal lineage ground cells into pavement cells, hence steering the division and growth of epidermal cells and modulating the stomatal density. In *SMR1*-overexpressing Arabidopsis plants under drought stress, the number of stomata per unit leaf area is decreased compared to the wild type, reducing transpiration and resulting in enhanced drought tolerance. In the absence of SMR1, *smr1* mutant plants fail to maintain a low stomatal density under drought conditions, resulting in enhanced wilting. However, the lowering of the stomata-mediated gas-exchange capacities of the leaves by high *SMR1* expression levels restrains photosynthesis, leading to stunted growth. Therefore, exploitation of *SMR1* overexpression to improve drought tolerance of crop plants is questionable. Enhancing drought resistance without compromising growth would require appropriate approaches such as drought-inducible, leaf epidermis-specific upregulation of *SMR1*.

Non-stomatal transpiration is another source of water losses in plants, and its modulation can impact drought tolerance (Yeats and Rose, 2013; Xue et al., 2017). Several parameters can affect non-stomatal transpiration such as the amounts of cutin and waxes deposited at the leaf surface or the density of trichomes. A necessary step in trichome development is endoreplication (Walker et al., 2000; Schnittger et al., 2003; Larkin et al., 2007). As explained above, endoreplication is an alternative form of the cell cycle where DNA replication is repeated without mitosis or cytokinesis, increasing DNA content and ploidy (Lang and Schnittger, 2020). Endoreplication is believed to be an adaptive, plastic response to attenuate the effects of stress (Carneiro et al., 2021). CKIs play a role in the endoreplication process by suppressing CDK activities (Shimotohno et al., 2021), and SIM was reported to be required for this mechanism in trichome development (Churchman et al., 2006). The morphology and density of trichomes help plants to adapt to different abiotic stresses such as salt, temperature and drought, by reducing transpiration and regulating leaf temperature (Chen et al., 2022). Consequently, SIM could constitute a target for improving plant tolerance to water stress and/or heat stress. Unfortunately, SIM-overexpressing Arabidopsis plants have been reported to have a dwarf phenotype, and their trichomes did not differ significantly from those of the wild type in size or degree of branching (Churchman et al., 2006), undermining this idea. Moreover, SIM expression is not very sensitive to environmental constraints (Yi et al., 2014), suggesting that its primary function is not the resistance to abiotic stresses.

*SMR5* is the most sensitive *SMR* gene to environmental stresses. In particular, *SMR5* expression is strongly stimulated by drought stress conditions, much more than the expression of the other *SMR*s including *SMR1* (Yi et al., 2014; Dubois et al., 2018; Braat et al., 2023). SMR5 overexpression was recently found to markedly increase drought tolerance (Braat et al., 2023). Interestingly, SMR5-induced protection was not associated with changes in stomatal conductance and had a limited effect on shoot growth under optimal conditions. In contrast, high *SMR5* expression levels brought about a marked inhibition of root growth and pronounced physiological changes in root tissues, such as suberin accumulation and up-regulation of the cellular detoxification pathway. Moreover, many marker genes for water privation were constitutively induced in *SMR5*-overexpressing roots. Thus, high SMR5 levels are somehow perceived by the plant as a drought stress signal.

The YAK1 kinase is involved in TOR-dependent transcriptional regulation of *SMR* genes, including *SMR5*, hence impacting cell cycle and meristem activity (Barrada et al., 2019). Interestingly, the Arabidopsis *yak 1* mutant is more sensitive to drought than the wild type (Kim et al., 2016). However, this decreased drought tolerance of *yak1* was attributed to the role of YAK1 in ABA-mediated responses and in stomatal closure. This is not in line with the absence of effects of *SMR5* overexpression and β-CCA on the stomatal functioning (Braat et al., 2023), making the participation of YAK1 to the protective effects of SMR5 unlikely.

The global stimulation of stress defense mechanisms by SMR5 could make this protein a potential candidate for engineering drought stress-resilient plants. In a transcriptomic analysis of maize seedlings exposed to heat, cold, salt or drought, SMR was also associated with the response to abiotic stresses and with seed development (Zhang et al., 2020a). The concomitant effects on cell division and cell protection suggest a role for SMRs in the regulation of the growth-defense trade-off (**Figure 4**). Future strategies for resetting the balance between stress resistance and growth to engineer stress-resistant and high-yielding crops require the understanding of how stress signaling regulates plant growth (Zhang et al., 2020b). *SMR5* and its inducer β-CCA are new pieces of the puzzle which could possibly be used to develop one of these strategies.

## 6 Future directions

The role of SMRs in the response of plants to environmental stresses and in the balance between growth and stress defense is still largely unexplored. Many important questions remain to be investigated, which could constitute future research objectives:

- Is drought tolerance inducible by high SMR5 levels in crop plant species?
- What are the upstream steps in the SMR5-induced pathway leading to stress tolerance?
- What is the primary target of β-CCA and how the signal leads to induction of SMR5 expression?
- Why roots are more affected by SMR overexpression than the plant aerial parts? And how root growth inhibition is related to drought stress tolerance?
- Rather surprisingly, single and multiple *smr* mutants of Arabidopsis showed very little differences with the wild type under normal and stress conditions (Hendrix et al., 2020; Braat et al., 2023). Are there functional redundancies within the SMR family which could explain this lack of effects?
- High expression levels of *SMR5* and *SMR4* led to a marked enhancement of drought tolerance (Braat et al., 2023). Considering that the *SMR* gene family is large, do also other SMRs modulate plant tolerance to abiotic stresses?

Answering these questions will undoubtedly clarify the roles of the SMR family in plant stress physiology and will hopefully open new avenues for improving stress resilience of plants. This review focuses on the role of SMR cell-cycle inhibitors in the responses of plants to abiotic stresses. However, we would like to mention that CKIs could also play a role in biotic stress responses (Kumar and Larkin, 2017). Several observations point to a likely connection between CKIs and plant immune responses (Wang et al., 2014; Hamdoun et al., 2016). This aspect has been relatively understudied and promises to be an interesting and exciting area of research in the years to come.

## Author contributions

JB: Writing – original draft, Writing – review & editing. MH: Writing – original draft, Writing – review & editing.
